# Supplementary material for: Effect of Oat β-Glucan on Affective and Physical Feeling States in Healthy Adults: Evidence for Reduced Headache, Fatigue, Anxiety and Limb/Joint Pains
Source: Nutrients. 2021 May 1;13(5):1534. doi: 10.3390/nu13051534 (PMC8147290; doi:10.3390/nu13051534)
Supplement: Supplementary file 1 [file nutrients-13-01534-s001.zip › nutrients-1160800-supplementary.pdf]

Inclusion criteria

- fasting calculated LDL cholesterol between 3.00 and 5.00 mmol/L, inclusive
- males and non-pregnant, non-lactating females aged 18-65 yr inclusive
- willing and able to give informed consent
- agree to consume the investigational product three times daily for the duration of the study
- BMI of 18.5 to <40.0 kg/m2 (65-75%, or n=135-155 with a BMI<30; 12-25% or n=25-50 with a BMI 30.00-34.99; and 2-12% or n=5-25 with a BMI 35.00-39.99).
- Blood pressure <160/100 mmHg
- fasting triglycerides <4.0 mmol/L
- fasting serum glucose <126mg/dL (<7 mmol/L)
- serum urea and creatinine < 1.8 times upper limit of normal (ULN) unless approved by the qualified investigator (QI)
- serum aspartate aminotransaminase (AST), alanine aminotransferase (ALT) and gamma-glutamyl transferase (GGT) < 2 times ULN unless approved by QI
- hemoglobin > 0.9 times lower limit of normal and < 1.1 times ULN unless approved by QI
- willing to refrain from consumption of oat products, barley products and psyllium husk products during the intervention period
- willing to maintain their habitual diet
- have a stable body weight (<7 kg change) over the past 3 mo, and
- maintain current level of physical activity, habitual diet and body weight throughout the trial
- absence of health conditions that would prevent fulfillment of study requirements as judged by the Investigator on the basis of medical history
- understanding the study procedures and willing to provide informed consent to participate in the study and authorization to release relevant protected health information to the study investigator

Exclusion criteria

- failure to meet any one of the inclusion criteria
- participation in any PepsiCo trial within 6 mo of randomization
- participation in any clinical trial within 30 d of participation
- pregnancy, breastfeeding or planning to be pregnant
- allergy or sensitivity to study product ingredients
- dislike description of study product
- diet containing ≥15% of energy from saturated fat
- consuming >14 g fiber/1000 Kcal (including soluble fiber supplements)
- consumption of more than 2 drinks/d of alcohol, or more than 14 drinks/wk
- eating at unusual times in the opinion of the dietitian/nutritionist
- extreme dietary habits (i.e. Atkins diet, very high protein diets, etc.) in the opinion of the dietitian/nutritionist
- smoking >5 cigarettes per day, (no more than 15.5% of total smokers per arm)
- vaping (vaporizers, e-cigarettes, etc.); smokeless tobacco, and nicotine patch or gum use
- use of cholesterol lowering medication or prescription drug for treating diabetes mellitus within 4 wk of randomization
- active major gastrointestinal disorder
- travel outside of Canada / USA within 2 wk of screening; travel away from home for more than 3 consecutive nights during the study period
- major trauma or hospitalization for a medical condition or major surgical event within 6 mo of randomization
- history of cancer within two yr of randomization, except for non-melanoma skin cancer

- any condition or substance use which might, in the opinion of the PI, make participation dangerous for the participant, lead to poor attendance or compliance or affect the results

Withdrawal criteria

- Withdrawal of consent for any reason
- Less than 90% compliance (38/42 sachets every 2 wk) with test product consumption based on sachet count, failure to avoid consumption of oat products or other β-glucan rich products such as barley and psyllium to a maximum of 3 servings/wk, or to maintain their habitual (low fat and low fiber) diet, in the opinion of the study staff
- During the course of the study, the development of an injury or illness or initiation of use of a medication which, in the opinion of Dr. Wolever, makes the subject's continued participation dangerous to the subject or to others, or which may affect the results.
- Failure to follow INQUIS safety guidelines
- Repeated failure to attend at scheduled visits
- Repeated failure to follow the protocol

Table S1: Nutritional composition of intervention products (per sachet)

	Active Intervention	Control Intervention
Weight (g)	21.0	20.2
Energy (kcal)	77	74
Total fat (g)	0.9	0.9
Saturated fat (g)	0.0	0.0
Total carbohydrate (g)	15.4	16.4
Total sugars (g)	9.3	8.7
Added sugars (g)	9.0	8.5
Starch (g)	4.2	5.4
Total fiber (g)	1.9	0.3
Sugar Alcohol (g)	0.0	2.1
Avail. Carbohydrate (g)	13.5	14.1
B-glucan (g)	1.0	0.0
Protein (g)	1.9	1.9
Ingredients:	Hydrolyzed Oat Bran, sugar, maltodextrin, silicon dioxide, natural flavor.	Sugar, hydrolyzed brown rice flour, erythritol, pea protein concentrate, powdered shortening (high oleic sunflower oil, maltodextrin, food starch modified, sodium silicoaluminate, mixed tocopherols), cellulose gum, silicon dioxide, natural flavors, mixed tocopherols.

Table S2: Occurrence of 8 of the 16 non-gastro-intestinal symptoms on the symptoms questionnaire.

Symptom	Visit	Control (n=95)				Test(n=96)			
		None	Mild	Mod	Severe	None	Mild	Mod	Severe
Headache	Screen 2	69	20	5	1	73	21	2	0
	Baseline	72	18	4	1	76	15	5	0
	Week 2	65	18	11	1	75	17	4	0
	Week 4	67	23	4	1	77	13	5	1
Fatigue	Screen 2	61	26	7	1	56	38	2	0
	Baseline	67	26	2	0	57	35	4	0
	Week 2	65	27	3	0	64	29	2	1
	Week 4	65	29	1	0	71	22	2	1
Feelings of anxiety	Screen 2	76	15	4	0	70	26	0	0
	Baseline	76	18	1	0	77	17	2	0
	Week 2	79	13	3	0	81	13	2	0
	Week 4	79	15	1	0	83	11	2	0
Pains in joints or limbs	Screen 2	65	21	7	2	66	24	5	1
	Baseline	73	17	3	2	74	21	1	0
	Week 2	74	11	7	3	74	20	2	0
	Week 4	69	18	6	2	73	21	2	0
Tend to become exhausted	Screen 2	72	16	7	0	76	20	0	0
	Baseline	77	17	1	0	79	16	1	0
	Week 2	82	10	3	0	89	6	1	0
	Week 4	81	14	0	0	84	11	1	0
Lack of energy	Screen 2	67	20	8	0	59	36	1	0
	Baseline	72	19	4	0	72	20	4	0
	Week 2	75	18	1	1	78	16	1	1
	Week 4	76	17	2	0	79	14	2	1
Lack of appetite	Screen 2	84	10	1	0	89	7	0	0
	Baseline	85	9	1	0	88	8	0	0
	Week 2	77	17	1	0	87	7	1	1
	Week 4	82	12	1	0	85	9	1	1
Increased appetite	Screen 2	85	9	1	0	87	7	2	0
	Baseline	90	4	1	0	89	6	1	0
	Week 2	85	10	0	0	89	7	0	0
	Week 4	89	6	0	0	92	4	0	0

Values are numbers of participants with no symptom (None), or rating the symptom as Mild, Moderate (Mod) or Severe.

Table S3: Occurrence of the remaining 8 of the 16 non-gastro-intestinal symptoms on the symptoms questionnaire.

Symptom	Visit	Control (n=95)				Test(n=96)			
		None	Mild	Mod	Severe	None	Mild	Mod	Severe
Palpitation / Throbbing of heart	Screen 2	89	4	2	0	90	6	0	0
	Baseline	89	6	0	0	94	2	0	0
	Week 2	87	7	1	0	92	4	0	0
	Week 4	91	3	1	0	92	4	0	0
Diminished ability to concentrate	Screen 2	80	14	1	0	80	16	0	0
	Baseline	82	13	0	0	89	6	1	0
	Week 2	85	10	0	0	89	6	1	0
	Week 4	84	10	1	0	89	7	0	0
Balance disturbances	Screen 2	90	4	1	0	94	1	1	0
	Baseline	91	4	0	0	91	5	0	0
	Week 2	92	3	0	0	93	2	1	0
	Week 4	91	4	0	0	91	5	0	0
Feeling cold	Screen 2	79	14	2	0	78	17	1	0
	Baseline	81	13	1	0	81	14	1	0
	Week 2	86	8	1	0	84	11	1	0
	Week 4	82	13	0	0	90	6	0	0
Hot flashes / sensation of rising heat	Screen 2	75	15	4	1	79	11	6	0
	Baseline	80	12	2	1	76	15	4	1
	Week 2	79	11	4	1	79	12	5	0
	Week 4	79	13	2	1	81	13	2	0
Gloomy thoughts	Screen 2	81	13	1	0	83	12	1	0
	Baseline	89	6	0	0	86	9	1	0
	Week 2	85	10	0	0	88	7	1	0
	Week 4	88	6	1	0	88	6	2	0
Inner tension	Screen 2	78	15	2	0	76	19	1	0
	Baseline	88	6	1	0	84	10	1	1
	Week 2	88	6	1	0	87	9	0	0
	Week 4	87	8	0	0	86	8	2	0
Numbness / burning / itching hands feet	Screen 2	88	7	0	0	87	8	1	0
	Baseline	88	7	0	0	88	7	1	0
	Week 2	91	4	0	0	92	4	0	0
	Week 4	90	5	0	0	91	5	0	0

Values are numbers of participants with no symptom (None), or rating the symptom as Mild, Moderate (Mod) or Severe.

Table S4: Effect of sex, age and BMI on symptoms at Baseline.

Symptom	Endpoint	Sex[1]		Age[2]				BMI[3]				
		F (119)	M (72)	Young (107)	Old (84)	Mean (SD) None	Mean (SD) Any	Lean (60)	OW (71)	OB (60)	Mean (SD) None	Mean (SD) Any
Headache	Presence	27	17	20	25	47.7 (11.5)	47.2 (11.4)	22	24	23	28.0 (4.6)	28.2 (5.0)
	Mod/Sev	28	8	18	26			25	18	36		
Fatigue	Presence	40	25	25	42	46.1 (11.5)	50.6 (10.8)	33	32	38	28.0 (4.7)	28.0 (4.7)
	Mod/Sev	13	0	11	5			10	4	13		
Limb/Joint Pain	Presence	29	11	11	32	46.3 (11.5)	52.3 (9.8)	17	20	32	27.9 (4.7)	29.1 (4.8)
	Mod/Sev	14	13	11	15			0	0	32		
Anxiety	Presence	24	14	20	21	47.8 (11.3)	46.9 (11.9)	23	17	22	28.0 (4.5)	28.0 (5.1)
	Mod/Sev	7	10	12	5			0	0	5		
Lack of Energy	Presence	27	19	23	25	47.5 (11.9)	48.0 (9.9)	18	23	32	27.7 (4.5)	28.9 (5.0)
	Mod/Sev	25	0	11	22			27	13	16		
Exhaustion	Presence	19	15	18	18	47.6 (11.4)	47.7 (11.6)	12	18	23	27.8 (4.6)	29.0 (5.0)
	Mod/Sev	9	0	7	5			0	8	7		
Lack of Appetite	Presence	10	7	8	9	47.7 (11.6)	47.2 (10.1)	5	9	13	27.8 (4.6)	29.3 (5.0)
	Mod/Sev	8	0	0	10			0	17	0		
Gloomy Thoughts	Presence	10	6	8	8	47.6 (11.5)	48.4 (11.1)	5	10	10	27.8 (4.6)	29.5 (5.1)
	Mod/Sev	8	0	14	0			0	0	17		
Inner Tension	Presence	11	7	8	10	47.6 (11.3)	48.1 (12.5)	7	9	13	27.8 (4.6)	29.7 (5.3)
	Mod/Sev	15	20	29	9			0	0	38		
Reduced Ability to Concentrate	Presence	12	8	8	12	47.6 (11.7)	48.1 (9.4)	10	10	12	27.9 (4.7)	28.3 (4.2)
	Mod/Sev	7	0	14	1			0	0	14		
Hot Flashes	Presence	27	4	7	27	46.6 (11.4)	52.0 (10.5)	18	18	18	27.9 (4.6)	27.7 (4.5)
	Mod/Sev	19	67	33	21			9	38	18		
Feeling Cold	Presence	6	20	10	19	47.2 (11.2)	50.3 (12.3)	18	11	15	28.0 (4.6)	27.4 (4.8)
	Mod/Sev	8	0	13	5			18	0	0		

Values under "Sex (F/M)", "Age (Young/Old)" and "BMI (Lean/OW/OB)" are the percent of participants who, at the Baseline visit, rated the symptom as being present (Presence = either mild, moderate or severe) and the percent of participants in whom the symptom was present at Baseline who rated it as being moderate or severe (Mod/Sev) (eg. 27% of 119 Females (n=32) had headache at baseline and 28% of those (n=9) rated it as being moderate or severe).

Values under "Age" and "BMI" headed "Mean (SD)" are the mean and SD age (yr) or BMI (kg/m²) of participants who, at Baseline, rated the symptom as being absent (None) or present (Any of either mild, moderate or severe).

Sex, F=female, M=Male; Age, Young = <48 yr, Old = ≥48 yr; BMI, Lean = BMI<25 kg/m², OW = overweight (BMI 25 to 29.99 kg/m² inclusive), OB = obese (BMI ≥30 kg/m²). Under each of these column headings the number of participants is shown in brackets.

[1] Shaded values differ significantly by chi-squared test (p<0.05, p<0.01).
[2] Shaded values differ significantly by chi-squared test or 2-tailed t-test (p<0.05, p<0.01).
[3] Shaded values differ significantly by chi-squared test (p<0.05).

Table S5: Severity of symptoms when compared to severity at the Baseline visit.

		Week 2			Week 4			Mean Weeks 2&4		
		More	Less	p[1]	More	Less	p[1]	More	Less	p[1]
Headache	Test	11	11	0.077	10	13	0.16	19	13	0.20
	Control	17	6*		13	8		21	12	
Fatigue	Test	6	11	0.14	5	19*	0.021	9	18	0.057
	Control	9	7		12	9		17	13	
Limb/Joint Pain	Test	5	4	0.32	7	5	0.28	10	7	0.26
	Control	9	7		9	4		13	9	
Anxiety	Test	1	6	0.11	2	9*	0.27	2	9*	0.11
	Control	8	8		4	8		9	12	
Lack of Energy	Test	7	11	0.27	6	13	0.28	11	15	0.22
	Control	7	12		5	13		11	16	
Exhaustion	Test	4	13*	0.23	4	7	0.32	7	14	0.27
	Control	5	8		3	8		6	11	
Lack of Appetite	Test	7	3	0.32	10	4	0.34	14	4*	0.29
	Control	12	3*		6	3		14	5*	
Gloomy Thoughts	Test	0	2	0.13	2	3	0.32	2	4	0.13
	Control	2	3		4	2		8	3	
Inner Tension	Test	3	7	0.36	4	6	0.42	6	7	0.35
	Control	2	2		2	2		3	2	
Reduced Ability to Concentrate	Test	3	3	0.34	2	3	0.40	4	4	0.33
	Control	3	6		5	6		5	7	
Hot Flashes	Test	4	7	0.16	2	10*	0.098	5	10	0.13
	Control	8	5		5	5		9	7	
Feeling Cold	Test	5	7	0.28	1	9*	0.070	4	10	0.19
	Control	4	9		6	7		8	11	

Values are the % of participants in whom the symptom was more severe or less severe than the severity at the Baseline visit out of n=96 on Test and n=95 on Control.

* Ratio of M:L significantly different from 1:1 (p<0.05 by binomial distribution) where M and L are the number of participants in whom the symptom was more severe (M) or less severe (L).

[1] P-values are the significance of the difference between the ratio of M:L on Test vs Control by Fisher's exact test). Light orange shading shows differences with p<0.10, and yellow shading shows differences with p<0.05.
